# Primary coenzyme Q10 deficiency-7: expanded phenotypic spectrum and a founder mutation in southern Chinese

**DOI:** 10.1038/s41525-019-0091-x

**Published:** 2019-08-05

**Authors:** Mullin Ho-Chung Yu, Mandy Ho-Yin Tsang, Sophie Lai, Matthew Sai-Pong Ho, Donald M. L. Tse, Brooke Willis, Anna Ka-Yee Kwong, Yen-Yin Chou, Shuan-Pei Lin, Catarina M Quinzii, Wuh-Liang Hwu, Yin-Hsiu Chien, Pao-Lin Kuo, Victor Chi-Man Chan, Cheung Tsoi, Shuk-Ching Chong, Richard J. T. Rodenburg, Jan Smeitink, Christopher Chun-Yu Mak, Kit-San Yeung, Jasmine Lee-Fong Fung, Wendy Lam, Joannie Hui, Ni-Chung Lee, Cheuk‐Wing Fung, Brian Hon-Yin Chung

**Affiliations:** 1 Department of Paediatrics & Adolescent Medicine, LKS Faculty of Medicine, The University of Hong Kong, Hong Kong, China; 2 Department of Radiology, Queen Mary Hospital, Hong Kong, China; 3 Department of Pediatrics, National Cheng Kung University Hospital, Tainan, Taiwan; 4 Department of Pediatrics, MacKay Children’s Hospital, Taipei, Taiwan; 5 Department of Neurology, Columbia University Medical Center, New York, NY United States; 6 Department of Paediatrics and Medical Genetics, National Taiwan University Hospital and National Taiwan University College of Medicine, Taipei, Taiwan; 7 Department of Obstetrics and Gynecology, National Cheng-Kung University Hospital, Tainan, Taiwan; 8 Department of Paediatrics and Adolescent Medicine, Pamela Youde Nethersole Eastern Hospital, Hong Kong, China; 9 Pediatric Department, Macau CHCSJ Hospital, Macau, China; 10 Department of Paediatrics, Faculty of Medicine, The Chinese University of Hong Kong, Hong Kong, China; 11 Radboud Centre for Mitochondrial Medicine, Department of Paediatrics, Radboud Institute for Molecular Life Sciences, Radboud University Nijmegen Medical Centre, Nijmegen, The Netherlands; 12 Department of Paediatrics and Adolescent Medicine, Hong Kong Children’s Hospital, Hong Kong, China

**Keywords:** Diseases, Disease genetics

## Abstract

Primary coenzyme Q10 deficiency-7 (COQ10D7) is a rare mitochondrial disease caused by biallelic mutations in *COQ4*. Here we report the largest cohort of COQ10D7 to date, with 11 southern Chinese patients confirmed with biallelic *COQ4* mutations. Five of them have the classical neonatal-onset encephalo-cardiomyopathy, while the others have infantile onset with more heterogeneous clinical presentations. We also identify a founder mutation *COQ4* (NM_016035.5): c.370G>A, p.(Gly124Ser) for COQ10D7, suggesting a higher chance of occurrence in the southern Chinese. This study helps improve understanding of the clinical spectrum of this disorder.

## Introduction

Coenzyme Q10 (CoQ), also known as ubiquinone, is crucial for the function of mitochondrial respiratory chain complexes. Currently, the pathway of CoQ biosynthesis is known to involve at least 18 proteins. Primary CoQ deficiencies in humans involve genetic mutations in *COQ2*, *PDSS1*, *PDSS2*, *COQ8A*, *COQ9*, *COQ6*, *COQ4*, or *COQ7*.^1^

The primary CoQ10 deficiency-7 (COQ10D7, MIM: 616276) is caused by autosomal-recessive mutations in *COQ4*. *COQ4* is hypothesized to take part in stabilizing the CoQ complex.^2^ To date, biallelic *COQ4* mutations have been described in 16 patients from 11 unrelated families. They were reported in two case series (with five to six cases each) and three case reports. The patients described had common features of cardiomyopathy, encephalopathy, lactic acidosis often with a neonatal onset, and death in the neonatal or infantile period.^2–4^ Two siblings of childhood onset presenting with spinocerebellar ataxia and stroke-like episodes^5^ and two Chinese siblings with neonatal onset of dystonia, seizures, lactic acidosis, and cerebellar atrophy were described in recent case reports.^6^

In this report, we have 11 patients (4 males and 7 females) from 9 unrelated families who were managed by the Medical Genetics division of the Department of Paediatrics and Adolescent Medicine at the University of Hong Kong and the Department of Medical Genetics and Pediatrics at the National Taiwan University Hospital in the period of 2014–2018. They presented with two overlapping phenotypes: the classical neonatal-onset encephalo-cardiomyopathy and infantile-onset encephalopathy with or without cardiomyopathy. They were diagnosed as COQ10D7 due to homozygous or compound heterozygous *COQ4* mutations. All genetic diagnoses were made by whole-exome sequencing (WES) except Patient 4, Patient 5, and Patient 11 due to their known family history and recognizable features of COQ10D7. More importantly, we have identified a Chinese-specific *COQ4* founder mutation in 10 subjects, 5 of whom are homozygous for that mutation.

## Case reports

**Fig. 1:**
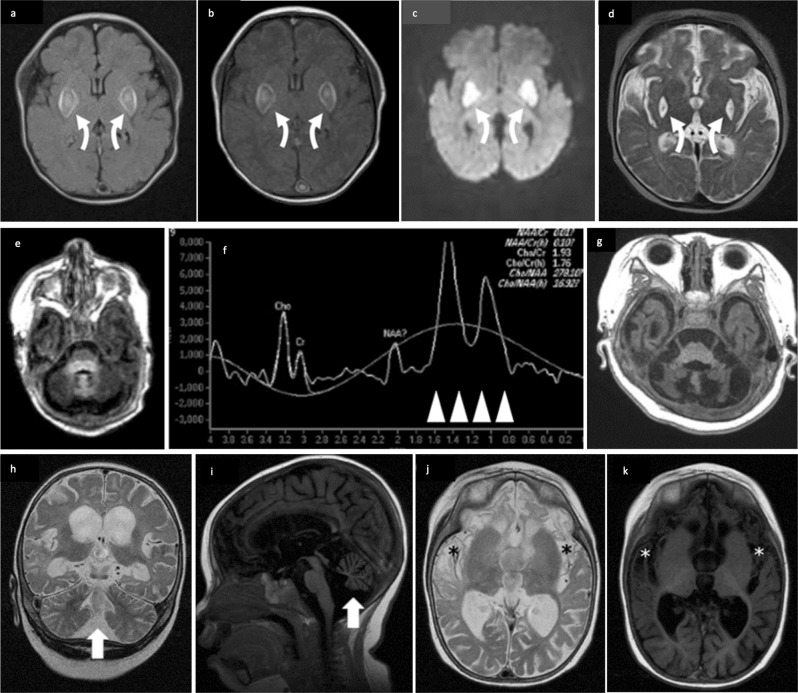
Cerebral magnetic resonance (MR) findings. **a** Axial T1W, **b** axial FLAIR, **c** DWI from Patient 1 at neonatal stage, and **d** axial T2W at follow-up; **e** axial T1W and **f** MR spectroscopy (MRS) at basal ganglia at neonate and **g** axial T1W at infant stages from Patient 4; **h** coronal T2W, **i** sagittal T1W, **j** axial T2W, **k** axial T1W from Patient 11. MR features include cerebellar atrophy (white arrows) (**h**, **i**) with progression (**e**, **g**); cerebral atrophy with frontal and anterior temporal lobar predominance (**i**–**k**); thinning of the corpus callosum (**i**); white matter loss and cystic change with frontal predominance (asterisks) (**j**, **k**); basal ganglia involvement with restricted diffusion and cystic change on follow-up (curved arrows) (**a**–**d**); lactate peak at around 1.3 ppm on MRS (arrowheads) (**f**)

**Fig. 2:**
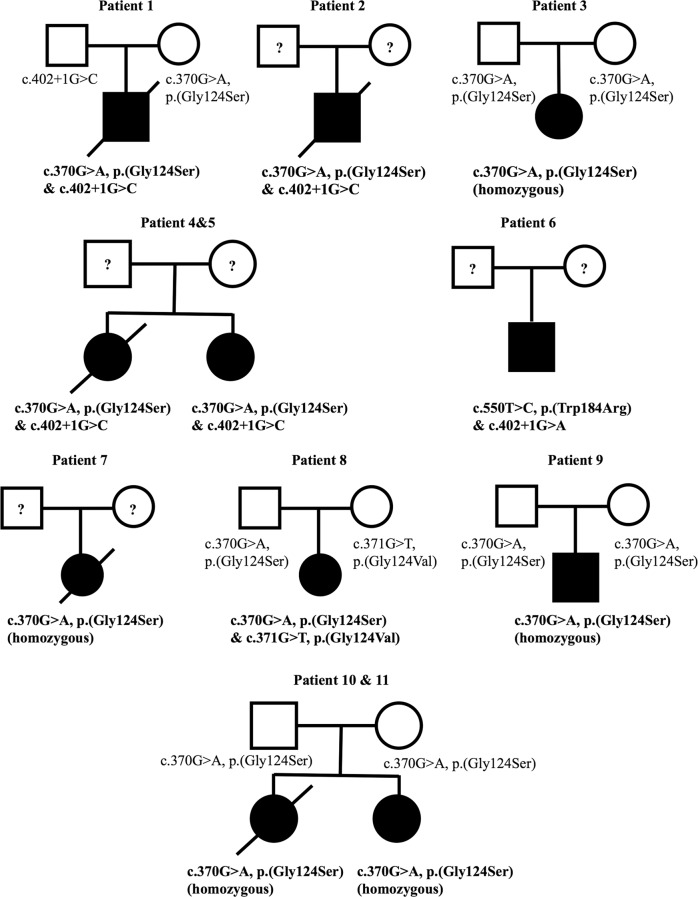
Pedigrees of 9 families with 11 subjects described in our study

The study was approved by the institutional review board of the University of Hong Kong/Hospital Authority Hong Kong West Cluster (UW12-211) and the National Taiwan University Hospital (201703073RINB). Written informed consent was obtained from subjects or their parents. A summary of the clinical, biochemical, and radiological characteristics of the 11 patients with *COQ4* mutations identified is presented in Table 1. Patients 1–5 had the classical neonatal-onset phenotype described by Brea-Calvo et al. and Chung et al.,^2,3^ whereas Patients 6–11 had later onset and more heterogeneous features. The frequency of distinct phenotypes compared with previous studies is summarized in Table 2. The corresponding MRI (magnetic resonance imaging) images are presented in Fig. 1. The pedigrees of the nine families are presented in Fig. 2.

**Table 1:** Summary of characteristics of 11 patients with *COQ4* mutations in this study and previously reported cases

Phenotype group	Neonatal-onset encephalo-cardiomyopathy									
Reference	This study					Brea-Calvo et al.^2^				
				Family 1				Family 3		
Subject	1	2	3	4	5	S1	S2	S3	S4	S5
Sex	Male	Male	Female	Female	Female	Male	Female	Female	Female	Male
Age at presentation	Neonatal	Neonatal	Neonatal	At birth	2 months	Neonatal	Neonatal	Neonatal	Neonatal	Infantile
Last follow-up	Passed away at 8 months (redirection of care)	Passed away at 2.5 days (unknown cause)	9 months	Now 4 years 6 months	Passed away at 1 year 1 month (respiratory failure)	Passed away at 4 h after birth	Passed away at 1 day	Passed away at 3 days	Passed away at 2 days	Still alive at age 18 years
*CoQ4* mutation	c.370G>A/c.402+1G>C	c.370G>A/c.402+1G>C	Homozygous c.370G>A	c.370G>A/c.402+1G>C	c.370G>A/c.402+1G>C	Homozygous c.433C>G	c.421C>T/c.718C>T	c.155T>C/c.521_523delCCA	c.155T>C/c.521_523delCCA	Homozygous c.190C>T
Hypotonia	√	×	√	√	√	√	×	√	×	×
Seizures	√	×	√	√	√	×	×	√	√	√
Cardiomyopathy	√	√	√	√	×	√	√	×	×	×
Other presented problems	Severe GDD, cortical visual impairment, bilateral severe to profound hearing impairment, myopathy	Apnea	Severe GDD	Severe DD, poor oromotor function	DD	Areflexia Acrocyanosis Respiratory failure Bradycardia	Severe IUGR Respiratory failure	Respiratory distress syndrome Distal arthrogryposis	Neonatal respiratory distress	Progressive motor deterioration after 10 months old, spastic ataxic gait at age 3 years Wheelchair bound by age 6 years Progressive swallowing difficulties requiring gastrostomy Cognitive deterioration Polyneuropathy with slow conduction Progressive scoliosis
Timing of MRI or other imaging	3 weeks and 3 months	—	7 weeks	7 days and 9 months	—	—	—	USG brain at birth	USG brain at birth	Age 12 and 17 years
MRI brain or other imaging findings	Symmetrical T1 and T2 hyperintensity with restricted diffusion at bilateral lentiform nuclei, subsequently infarcts with cystic changes. Foci of restricted diffusion also at bilateral frontal white matter. MRS: raised lactate peaks at bilateral basal ganglia and cerebral white matter. Mild cerebellar hypoplasia	—	Mild cerebellar hypoplasia, mild thinning of corpus callosum	Neonatal stage: symmetrical T1 hyperintensity at bilateral basal ganglia, mild cerebellar hypoplasia, later with generalized progressive cerebellar and cerebral atrophy with diffuse white matter loss, thinning of corpus callosum. Cystic changes within cerebral white matter, bilateral basal ganglia, thalami. MRS: raised lactate peaks at bilateral basal ganglia	—	—	—	USG brain: cerebellar hypoplasia Autopsy: Severe olivopontocerebellar and thalamic hypoplasia and scattered cavitations in the white matter	USG brain: cerebellar hypoplasia	MRI at age 12 years: bilateral increased signal intensity in FLAIR and T2W sequencing in both occipital cortical and juxtacortical areas MRI at age 17 years cerebellar atrophy, widened ventricular space, scars from cortical necrotic lesions in both occipital areas
Lactic acidosis	√	√	√	√	√	√	√	√	√	×
Effect of CoQ10 supplement	No significant improvement	No significant improvement	Cardiac function stable	No significant improvement	No significant improvement	Not used	Not used	Not used	Not used	Not used

Phenotype group	Neonatal-onset encephalo-cardiomyopathy										
Reference	Chung et al.^3^						Sondheimer et al.^4^	Bosch et al.^5^		Lu et al.^6^	
Subject	Family 1			Family 3				Family 1		Family 1	
	Proband 1	Sibling of Proband 1	Proband 2	Proband 3	Sibling of Proband 3	Proband 4	Patient	Patient 1	Patient 2	Patient II-1	Patient II-2
Sex	Female	Female	Female	Female	Female	Female	Male	Male	Female	Male	Female
Age at presentation	Neonatal	Neonatal	Neonatal	Neonatal	Neonatal	Neonatal	Neonatal	Diagnosed at age 13	8 months	2 months	2 months
Last follow-up	Passed away at 2 months (redirection of care)	Passed away at 4 days	Passed away at 4 days	Passed away at 19 months	Passed away at 10 weeks	Passed away at 7 weeks	Passed away at 4 months	—	3 years 7 months (oversea adoption)	Passed away at 5 months	3 years and 8 months
*CoQ4* mutation	c.245T>A/c.473G>A	Homozygous c.718C>T	Homozygous c.718C>T	c.197_198delGCinsAA/c.202G>C	c.197_198delGCinsAA/c.202G>C	Homozygous c.718C>T	c.23_33del11/c.331G>T/c.356C>T	Homozygyous c.230C>T	c.550T>C/c.402+1G>A	Homozygous c.370G>A	Homozygous c.370G>A
Hypotonia	√	—	√	√	√	√	√	—	—	×	×
Seizures	√	√ (Suspected)	×	√	×	×	√	√	√	√	√
Cardiomyopathy	√	√	√	√	×	√	√	—	—	— (cardiomegaly on chest X ray)	×
Other presented problems	—	IUGR Moderate cerebellar hypoplasia	IUGR Moderate cerebellar hypoplasia	Feeding difficulties, GDD, microcephaly	Feeding difficulties	Feeding difficulties, left hip dysplasia	Gastroesophageal reflux requiring fundoplication, delayed visual maturation without structural abnormality of the eyes, bilateral hearing loss, and absence of development	Delayed speech Tremor since age 4 years Progressive motor deterioration; wheelchair bound by age 12 years, then dysarthria Spastic tetraparesis Ataxia of upper and lower limbs Abnormal cognitive development	Tremor since age 10 years Moderate intellectual disability Frequent falls Right hemi-anopsia Second stroke-like episode at age 14 years	Dystonia since birth Hearing impairment Feeding difficulty Progressive motor deterioration Failure to thrive	Dystonia since birth Hearing impairment Feeding difficulty Progressive motor deterioration Nystagmus at 2 months
Timing of MRI or other imaging	Early neonatal period	—	—	Day 1	Fetal MRI at 30 weeks and Day 2	Unknown	First week and tenth week	5 years	10 years 13 years	2 months	1 month, 4 months and 3 years and 8 months
MRI brain or other imaging findings	Small cerebellar size and diffuse T2 white matter hyperintensity MRS decreased *N*-acetylaspartate and a lactate peak	Autopsy: Cerebellar and brainstem hypoplasia and microdysgenesis	Autopsy: Cerebellar and brainstem hypoplasia and microdysgenesis	Cerebellar hypoplasia, prominent extra axial space in posterior fossa and mild lateral ventricle enlargement	Fetal MRI: normal intracranial anatomy, transverse cerebellar diameter 10–15th percentile MRI on Day 2: decreased cerebellar hemisphere volume	Normal	First week: focal regions of cortical increased T1 signal and magnetic resonance spectroscopy identified enlarged lactate peaks Tenth week: microcephaly with volume loss and increasing prominence of lactate peaks	Suspected tectal glioma (treated with radiotherapy)	Age 10 years: Cavernoma in the left parietal lobe Age 13 years: Lesion at left occipital lobe with clear diffusion restriction	Slightly widened frontal and temporal lobes	1 month: normal 4 months: found lesions in midbrain and basal ganglia 3 years and 8 months: CT brain: symmetrical, patchy, low density shadow in bilateral basal ganglia and diffuse brain atrophy
Lactic acidosis	√	√	√	×	√	×	√	—	—	√	√
Effect of CoQ10 supplement	Improvement after 2 weeks of age but recurrent episode of metabolic and hemodynamic decompensation	Not used	Not used	Not used	Not used	No significant improvement	Not used	Walk test stable over the period of a year	Walk test stable over the period of a year	No significant improvement	Improved seizure control but no improvement in dystonia and motor development

Phenotype group	Infantile-onset encephalo-cardiomyopathy					
Reference	This study					
					Family 2	
Subject	6	7	8	9	10	11
Sex	Male	Female	Female	Male	Female	Female
Age at presentation	8 months	Since early infancy	Since early infancy	2 months	2 months	4 months
Last follow-up	3 years 7 months (overseas adoption)	Passed away at 3 years 6 months (unknown cause)	3 years 3 months	8 years	1 year 6 months	Passed away at 1 year 8 months (septic shock)
*CoQ4* mutation	c.550T>C/c.402+1G>A	Homozygous c.370G>A	c.370G>A/c.371G>T	Homozygous c.370G>A	Homozygous c.370G>A	Homozygous c.370G>A
Hypotonia	√	×	×	×	√	√
Seizures	×	×	√	√	√	√
Cardiomyopathy	×	×	×	×	√	√
Other presented problems	Severe GDD, generalized dystonia, cortical visual impairment, impaired oromotor function	Severe GDD, generalized dystonia and spasticity, cortical visual impairment, impaired oromotor function	Severe GDD, generalized dystonia and spasticity, cortical visual impairment	DD	Severe DD, bilateral cortical blindness	DD, intermittent spasticity, impaired oromotor function
Timing of MRI or other imaging	Day 21, Day 40, and 1 year 4 months	6 months	6, 7, and 35 months	32 months	14 months	1 year 2 months
MRI brain or other imaging findings	Mild cerebral atrophy with bilateral frontal predominance	Severe cerebral atrophy	Mild cerebral and cerebellar hypoplasia. Small focus of T2 and FLAIR hyperintensity at the left lentiform nucleus at 35 months. MRS: raised lactate peaks at bilateral basal ganglia and frontal white matter at 6 months, normalized by 7 months	Moderate cerebellar atrophy without isolated vermian hypoplasia, cerebral atrophy, symmetrical loss of cerebral white matter particularly in bilateral frontal and anterior temporal regions. Corpus callosum was thinned, basal ganglia and pons unremarkable	Mild thinning of corpus callosum	Mild cerebellar atrophy and cerebral atrophy, white matter cystic changes with bilateral frontal and anterior temporal predominance. Corpus callosum thinning, preserved basal ganglia and brainstem
Lactic acidosis	√	√	√	√	√	×
Effect of CoQ10 supplement	No significant improvement	No significant improvement	Subjective improvement in response	Stable condition	Some improvement in seizure control and development	Not used

*MRS* magnetic resonance spectroscopy, *MRI* magnetic resonance imaging, *FLAIR* fluid-attenuated inversion recovery, *GDD* global developmental delay, *DD* developmental delay, *IUGR* intrauterine growth restriction, *USG* ultrasound

**Table 2:** Phenotypic comparison between patients in this study and previously reported cases

	This study	Chung et al.^3^	Brea-Calvo et al.^2^	Sondheimer et al.^4^	Bosch et al.^5^	Lu et al.^6^
Number of subjects	11	6	5	1	2	2
Female-to-male ratio	7:4	6:0	3:2	0:1	1:1	1:1
Age of presentation	Birth to 8 months	Birth to day 1	Birth to 6 h	1 day	4–9 years	1–2 months
Neonatal onset	5/11 (45%)	6/6 (100%)	4/5 (80%)	1/1 (100%)	0/2 (0%)	2/2 (100%)
Infantile onset	6/11 (54%)	0/6 (0%)	1/5 (20%)	0/1 (0%)	0/2 (0%)	0/2 (0%)
Childhood onset	0/11 (0%)	0/6 (0%)	0/5 (0%)	0/1 (0%)	2/2 (100%)	0/2 (0%)
Respiratory distress	5/11 (45%)	6/6 (100%)	^a^4/4 (100%)	1/1 (100%)	—	2/2 (100%)
Cardiomyopathy	6/11 (54%)	5/6 (83%)	2/5 (40%)	1/1 (100%)	—	1/2 (50%)
Hypotonia	7/11 (64%)	^a^5/5 (100%)	2/5 (40%)	1/1 (100%)	—	—
Dystonia	2/11 (18%)	—	—	—	—	2/2 (100%)
Seizures	8/11 (73%)	3/6 (50%)	3/5 (60%)	1/1 (100%)	2/2 (100%)	2/2 (100%)
Lactic acidosis	10/11 (91%)	4/6 (67%)	4/5 (80%)	1/1 (100%)	—	2/2 (100%)
Cerebellar atrophy	6/11 (54%)	^a^4/5 (80%)	3/5 (60%)	—	—	2/2 (100%)
Basal ganglia	5/11 (45%)	—	—	—	—	1/2 (50%)

^a^Lacking information from one patient

### Patient 1

Patient 1 was a male with an antenatal history of oligohydramnios and intrauterine growth restriction (IUGR). He was born at 38 weeks. At 7 days old, he developed recurrent episodes of apnea, decreased activity, and mild lactic acidemia. On day 14, he developed circulatory collapse with severe metabolic acidosis and lactic acidosis up to 28.36 mmol/L (reference range 0.5–2.20), hypotension requiring multiple inotropes, an episode of pulseless ventricular tachycardia, and respiratory failure requiring intubation and ventilator support. Thereafter, he developed recurrent convulsions controlled by phenobarbitone and levetiracetam. MRI of the brain at 3 weeks of age showed symmetrical T1 and T2 hyperintensity with restricted diffusion at bilateral lentiform nuclei. Foci of restricted diffusion were also detected at bilateral frontal white matter (Fig. 1a–c). Magnetic resonance spectroscopy (MRS) showed raised lactate peaks at bilateral basal ganglia and cerebral white matter. Subsequent follow-up MRI showed established infarcts with cystic changes at bilateral lentiform nuclei (Fig. 1d). Mild cerebellar hypoplasia was also noted. Serial echocardiography in the following months showed progressive septal and ventricular myocardial hypertrophy. WES identified compound heterozygous mutations in *COQ4*, a missense c.370G>A, p.(Gly124Ser) and a splicing mutation c.402+1G>C. Functional analysis of the skin fibroblasts showed ETC complex II+III deficiency due to low CoQ concentration. At 5 months of age, CoQ10 supplement up to 40 mg/kg/day. At 8 months, in view of poor neurological prognosis and poor response to CoQ10 treatment, he was diverted to comfort care and extubated. He passed away shortly from respiratory failure.

### Patient 2

Patient 2 was a term newborn male. He developed respiratory distress, hypotension, and progressive metabolic acidosis with a lactate level of 2.6 mmol/L and hyperalaninemia requiring intubation and inotropic support on day 1 of life. Echocardiogram revealed hypertrophic cardiomyopathy. Plasma amino acids revealed high alanine, proline, and tyrosine, and acylcarnitine profile was unremarkable. CoQ10 supplement at 15 mg/kg/day and carnitine at 100 mg/kg/day were tried on his second day of life but the patient succumbed. WES revealed compound heterozygous mutations in *COQ4*: a missense c.370G>A, p.(Gly124Ser) and a splicing mutation of c.402+1G>C.

### Patient 3

Patient 3 was born full term at 37 weeks. She developed transient respiratory distress shortly after birth. On day 22 of life, she had cardiogenic shock. Echocardiogram showed poor contractility with a left ventricular ejection fraction of 20% and a moderate pericardial effusion. There was associated lactic acidemia (24 mmol/L; reference range 0.5–2.2) and hyperammonemia (139 µmol/L; reference range <100). She was empirically given CoQ10 supplementation and intravenous immunoglobulin. Her cardiac function improved gradually and normalized by day 32 of life. She developed seizures at 4 months of age requiring multiple anticonvulsants. She is severely delayed developmentally. WES revealed a homozygous *COQ4* mutation c.370G>A, p.(Gly124Ser).

### Patient 4 and Patient 5

Patient 4 was the younger sister of Patient 5. She had antenatal history of IUGR and was born at 38 weeks. Immediately postnatal, she developed respiratory distress with intermittent apnea and lactic acidemia (up to 10 mmol/L; reference range 0.5–2.2). MRI brain showed symmetrical T1 hyperintensity at bilateral basal ganglia, with mild cerebellar hypoplasia (Fig. 1e). MRS showed raised lactate peaks at bilateral basal ganglia (Fig. 1f). She developed treatment-resistant seizures at 2 months of age. Subsequent MRI at 9 months of age showed generalized progressive cerebellar and cerebral atrophy, with diffuse white matter loss including thinning of the corpus callosum. Cystic changes were seen within the cerebral white matter, bilateral basal ganglia, and thalami (Fig. 1g). Serial echocardiogram showed progressive moderate left ventricular hypertrophy. Owing to the recognizable clinical presentation, Sanger sequencing was performed and revealed compound heterozygous *COQ4* mutation: c.370G>A, p.(Gly124Ser) and c.402+1G>C. CoQ10 supplement has been started since age of 4 years 5 months.

Patient 5 was the elder sister of Patient 4. She was born at 39 weeks with an antenatal history of IUGR. She developed seizures from 2 months of age with associated lactic acidosis and respiratory failure requiring home ventilation. Chest radiograph showed cardiomegaly. CoQ10 supplementation was tried at 1 year of age but passed way from respiratory failure 1 month after. After the genetic diagnosis of her younger sister, Sanger sequencing was performed retrospectively and revealed the same compound heterozygous *COQ4* mutation as her sister, c.370G>A, p.(Gly124Ser) and c.402+1G>C.

### Patient 6

Patient 6 presented at 8 months of life with severe global developmental delay, microcephaly, generalized dystonia, cortical visual impairment, and oromotor dysfunction. Metabolic workup revealed lactic acidemia of 2.5–5.9 mmol/L and hyperalanemia (626 µmol/L; reference range 143–439). WES revealed compound heterozygous mutations in the *COQ4* gene: c.550T>C, p.(Trp184Arg) and c.402+1G>A. Functional analysis of the skin fibroblasts showed ETC complex II+III deficiency with low CoQ concentration. There was no further follow-up because of overseas adoption.

### Patient 7

Patient 7 was a girl, born full term. She had bilateral cortical visual impairment since birth and progressive oromotor dysfunction requiring gastrostomy feeding. She had severe global developmental delay. She developed generalized dystonia and spasticity around 5 months of age. Lactic acidemia of 2.4–3.2 mmol/L was present. WES revealed a homozygous variant in the *COQ4* gene: c.370G>A, p.(Gly124Ser). Functional analysis of the skin fibroblast showed ETC complex II+III deficiency and low CoQ concentration. She had been on CoQ10 supplement since 2 years old. There was no clinical improvement and the patient died at 3 years and 6 months of age.

### Patient 8

Patient 8 is a girl, born full term. She developed infantile spasms at 6 months of age. Metabolic workup showed lactic acidemia at 2.2–4.2 mmol/L and hyperalanemia (487 µmol/L; reference range 143–439). WES revealed compound heterozygous variants of the *COQ4* gene c.371G>T, p.(Gly124Val) inherited from the mother and c.370G>A, p.(Gly124Ser) inherited from the father. Interestingly, respiratory chain enzymology of the skeletal muscle activities was normal but skin fibroblast functional analysis showed ETC complex II+III deficiency and low CoQ10 concentration. CoQ10 supplement has been given since 9 months of age, with subjective improvement in responsiveness. She is alive and has achieved fair seizure control with levetiracetam and global developmental delay.

### Patient 9

Patient 9 is a boy, born full term at 40 weeks. He presented with infantile spasms at 2 months of age. CoQ10 supplementation started at 7 years of age and has remained stable. WES revealed a homozygous *COQ4* mutation, c.370G>A, p.(Gly124Ser). Skin fibroblast functional analysis showed ETC complex II+III deficiency and low CoQ concentration.

### Patient 10 and Patient 11

Patient 10 is the younger sister of Patient 11. She was born at 36 weeks. She developed transient respiratory distress after birth. She was asymptomatic until 2 months of age when she developed progressive hypotonia, cortical visual impairment, severe developmental delay, and seizures requiring multiple anticonvulsants. Her echocardiogram showed progressive dilated cardiomyopathy and mitral regurgitation. WES revealed a homozygous *COQ4* mutation: c.370G>A, p. (Gly124Ser). CoQ10 supplement at 30 mg/kg/day was started at 11 months of age, and her seizure control improved.

Patient 11 is the elder sister of Patient 10. She was born full term. At 4 months of age, she presented with seizures, hypotonia, spasticity, oromotor dysfunction, and severe developmental delay. She also developed an episode of acute myocarditis during which her echocardiogram showed diastolic dysfunction. Brain MRI at 14 months showed mild cerebellar atrophy and cerebral atrophy, white matter cystic changes with bilateral frontal and anterior temporal predominance, and thinning of the corpus callosum. Basal ganglia and brainstem appeared preserved (Fig. 1h–k). No lactic acidosis was detected. Owing to the exome findings of her sister, Sanger sequencing was performed and revealed a homozygous *COQ4* mutation: c.370G>A, p.(Gly124Ser). She was not on CoQ10 supplement and passed away at 20 months due to an episode of sepsis.

## Results

### Pathogenicity of the COQ4 variants

We analyzed the COQ4 variants identified in our cohort by previously reported literatures, ClinVar, population frequency in gnomAD,^7^ conversation score by Combined Annotation-Dependent Depletion,^8^ in silico prediction by Rare Exome Variant Ensemble Learner,^9^ and protein stability change prediction by STRUM^10^ (Table 3). All variants demonstrated a deleterious effect. Together with the reduced level of CoQ10 of the patients, the pathogenicity of these variants is strongly supported by the biochemical findings of the patients.

**Table 3:** Analysis of the four variants identified in our cohort

Variant		gnomAD population frequency	Reported to be disease causing?	CADD	REVEL	ddG
c.370G>A, p.(Gly124Ser)		1.13e−04	Yes (Lu et al.^6^)	24.8	0.817	−1.19
c.402+1G>C		2.79e−05	Yes (ClinVar)	28.8	N/A	N/A
c.371G>T, p.(Gly124Val)		3.98e−06	No	24.6	0.753	−1.53
c.550T>C, p.(Trp184Arg)		0	No	26.7	0.538	−0.62

*CADD* Combined Annotation-Dependent Depletion, *REVEL* Rare Exome Variant Ensemble Learner, *N/A* not available

### Founder mutation analysis

**Fig. 3:**
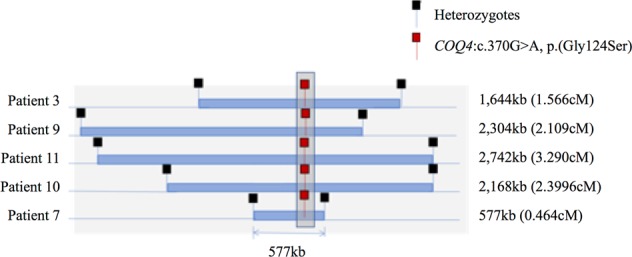
Founder mutation analysis. Shared haplotypes among homozygous COQ4:c.370G>A. Red square indicates the location of homozygous COQ4:c.370G>A, while black square indicates the nearest heterozygous single-nucleotide polymorphism. The length of the haplotype for each subject is at the right panel. The maximum shared length is approximately 577 kb

Among these 11 patients, we identified the same missense mutation c.370G>A, p.(Gly124Ser) in 10 of them. This missense mutation is a rare variant with a population frequency of 0.001118 and it is exclusively found in South East Asians in the gnomAD database.^7^ Further analysis of the DNA of the five homozygous patients using Infinium OmniZhongHua-8 BeadChip SNP array showed a common haplotype of 0.464–3.290 cM implying that the mutation was inherited from a common ancestor 27 generations ago (Fig. 3). In addition, by principal component analysis), our SNP array data are clustered with Chinese and Japanese of HapMap Phase II (*n* = 270) and a local southern Chinese database (*n* = 612). Furthermore, the pathogenicity of this mutation has been established via the reduced CoQ level in the skin fibroblasts in Patients 7 and 9 who have homozygous c.370G>A mutation (Table 4). Altogether our study confirms that the missense mutation c.370G>A, p.(Gly124Ser) represents a pathogenic founder mutation in the southern Chinese population.

**Table 4:** COQ level in the skin fibroblasts in patients with *COQ4* mutation

Patient	Tissue	COQ level	CI	CI+III	CII	CII+III	CIII	CIV
1	Skin fibroblast	0.4 pmol/U COX (1.64–3.32)	Normal	Not done	Normal	130 mU/U COX (269–781)	Normal	Normal
6	Skin fibroblast	0.63 pmol/U CS (1.04–2.92)	Normal	Not done	Normal	183 mU/U COX (269–781)	Normal	Normal
7	Skin fibroblast	0.4 pmol/UCOX (1.64–3.32)	Not done	Not done	Normal	183 mU/UCOX (269–781)	Normal	Not done
8	Muscle	191 pmol/mg (140–580)	Normal	Not done	Normal	Not done	Normal	Normal
	Skin fibroblast	0.29 nmol/UCOX (1.64–3.32)	Normal	Not done	Normal	135 mU/U COX (control 269–781 in the skin)	Normal	Normal
9	Skin fibroblast	16.4 ng/mg prot (46.1 ± 3)	Not done	64% of CS	90% of CS	55% of CS	Not done	67% of CS

Reference values are given in brackets. Experiment performed at the Radboud University Medical Centre, Nijmegen and the National Taiwan University Hospital

*CI* complex I, *CII* complex II, *CIII* complex III, *CIV* complex IV, *CS* citrate synthase

## Discussion

To our knowledge, this is the largest case series of primary COQ10D7 reported. In the literature, primary COQ10D7 cases have been described predominantly with a neonatal onset, with only two cases of childhood onset.^2–5,8^ In this study, we have expanded the phenotypic spectrum of primary COQ10D7 from neonatal to infantile onset.

We have five patients exhibiting the well-described neonatal presentation of COQ10D7 as in the literature, characterized by respiratory distress, encephalopathy, seizures, hypotonia, and cardiomyopathy. Previously, it was believed that phenotypes from affected males with *COQ4* mutation would be more severe and highly likely lethal.^3,6^ However, in our study the male-to-female death ratio was 2:3.

We have six patients with infantile-onset phenotypes. Unlike those of neonatal onset, MRI brain for those infantile-onset patients did not show characteristic basal ganglia lesions. Dystonia was observed in two out of the six patients with infantile-onset presentation in our cohort, and it was also observed in the two neonatal-onset cases reported by Lu et al.^8^ but not reported in non-Chinese patient.

**Fig. 4:**
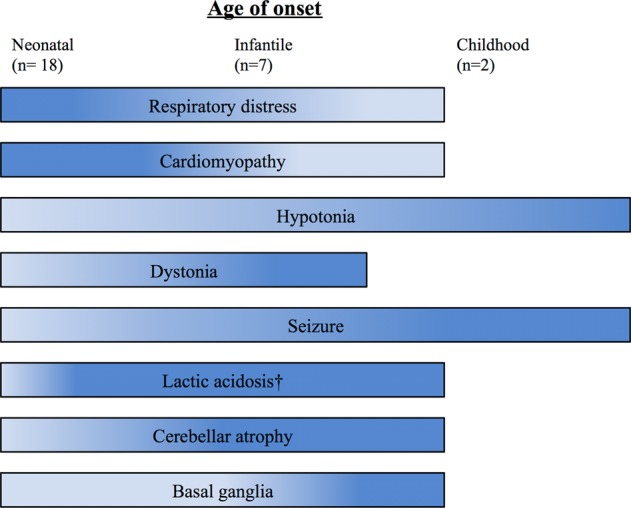
Phenotypic spectrum of neonatal, infantile, and childhood onset of COQ10D7 from all the reported cases including this study. †Lactic acidosis is not mentioned in the childhood-onset cases. (The color intensity representing the percentage of patients having that clinical presentation in arbitrary form)

A summary of the predominant phenotypes in the spectrum of neonatal, infantile, and childhood onset of COQ10D7 is shown in Fig. 4. The variegated symptoms and disease onsets explain the frequent delay of diagnoses of COQ10D7. This also highlights the importance of the complementarity of biochemical screening for children with unexplained neurological disturbances and the prompt application of WES in order to reach a genetic diagnosis that has an impact on patient management.

Functional analysis to demonstrate the deficiency of CoQ should be carefully examined because mitochondrial enzymology can be tissue specific. In this study, among the five patients (Patients 1, 6, 7, 8, 9) with ETC chain analysis in the skin fibroblast, all of them shown a reduced level of succinate:cytochrome *c* oxidoreductase (complex II+III). Measurement of CoQ level was also found significantly decreased. For Patients 7 and 8, complex II+III analysis and CoQ level measurement were also performed in the muscle. Interestingly, the CoQ level from the muscle is normal but that from skin fibroblasts was reduced. From the Genotype-Tissue Expression (GTex) data, the *COQ4* median expression in the muscle is 7.58 transcripts per million (TPM) while in the skin it is 44.14 TPM, demonstrating a 6-fold lower expression in the muscle.

More significantly, we have identified a common founder pathogenic *COQ4* mutation associated with COD10D7. In this study, 10 out of the 11 patients carry the *COQ4*: c.370G>A, p.(Gly124Ser) allele. This mutation fulfills the criteria of a founder mutation: (1) all patients with the mutant alleles share a haplotype associated with the mutation; (2) the haplotype is shared among affected families with a genetic distance >1 cM; (3) the mutant allele is rare and specific to the population; and (4) all carriers are delineated to the same geographic region. It is likely that this founder mutation causes a relatively higher rate of COQ10D7 in southern Chinese individuals, and that may explain why we can present a larger cohort as compared to past studies in this field.

CoQ10 oral supplementation was previously reported effective in *COQ4* mutation cases.^2–4,6^

Among the 10 patients who received CoQ10 supplement and with continuous follow-up, those shown with stabilized cardiac condition or seizure control are those of genotype of homozygous missense variant c.370G>A (Patients 3, 9, 10). Another patient on CoQ supplement with improved clinical condition is Patient 8 with genotype of compound heterozygous missense variants c.370G>A/c.371G>T. For those without improvement are patients with genotype in the presence of a splicing mutation c.402+1G>A (Patients 1, 2, 4, 5, 6). Among these five patients, three of them (Patients 1, 2, and 5) died from the disease. Retrospectively, Patients 1, 4, and 5 were documented IUGR antenatally. This may suggest that the presence of c.402+1G>A, a loss-of-function mutation, would cause more severe neonatal onset of phenotypes and less responsive to CoQ10 supplement.

In this study, we have expanded the phenotypic spectrum of *COQ4* mutation. Now COQ10D7 can range from neonatal, infantile to childhood onset. We have also identified a pathogenic *COQ4* founder mutation in the southern Chinese population. The importance of complementarity of biochemical screening and prompt application of WES on patients with unexplained neurological symptoms is highlighted.

### Reporting summary

Further information on experimental design is available in the Nature Research Reporting Summary linked to this paper.

## Supplementary information

Reporting Summary

## Data Availability

The data that support the findings in this study are available on request from the corresponding authors (N.-C.L., C.-W.F., B.H.-Y.C.). The data are not publicly available as they contain information that could compromise research participant privacy or consent.

## Supplementary information

**Supplementary Information** accompanies the paper on the *npj Genomic Medicine* website (10.1038/s41525-019-0091-x).

## References

[1] Desbats MA, Lunardi G, Doimo M, Trevisson E, Salviati L (2015). Genetic bases and clinical manifestations of coenzyme Q10 (CoQ 10) deficiency. J. Inherit. Metab. Dis..

[2] Brea-Calvo G (2015). COQ4 mutations cause a broad spectrum of mitochondrial disorders associated with CoQ10 deficiency. Am. J. Hum. Genet..

[3] Chung WK (2015). Mutations in COQ4, an essential component of coenzyme Q biosynthesis, cause lethal neonatal mitochondrial encephalomyopathy. J. Med. Genet..

[4] Sondheimer N (2017). Novel recessive mutations in COQ4 cause severe infantile cardiomyopathy and encephalopathy associated with CoQ10 deficiency. Mol. Genet. Metab. Rep..

[5] Bosch AM (2018). Coenzyme Q10 deficiency due to a COQ4 gene defect causes childhood-onset spinocerebellar ataxia and stroke-like episodes. Mol. Genet. Metab. Rep..

[6] Lu M (2019). Clinical phenotype, in silico and biomedical analyses, and intervention for an East Asian population-specific c.370G>A (p.G124S) COQ4 mutation in a Chinese family with CoQ10 deficiency-associated Leigh syndrome. J. Hum. Genet..

[7] Lek M (2016). Analysis of protein-coding genetic variation in 60,706 humans. Nature.

[8] Rentzsch P, Witten D, Cooper GM, Shendure J, Kircher M (2019). CADD: predicting the deleteriousness of variants throughout the human genome. Nucleic Acids Res..

[9] Ioannidis NM (2016). REVEL: an ensemble method for predicting the pathogenicity of rare missense variants. Am. J. Hum. Genet..

[10] Quan L, Lv Q, Zhang Y (2016). STRUM: structure-based stability change prediction upon single-point mutation. Bioinformatics.

